# Enhanced simulated early 21st century Arctic sea ice loss due to CMIP6 biomass burning emissions

**DOI:** 10.1126/sciadv.abo2405

**Published:** 2022-07-27

**Authors:** Patricia DeRepentigny, Alexandra Jahn, Marika M. Holland, Jennifer E. Kay, John Fasullo, Jean-François Lamarque, Simone Tilmes, Cécile Hannay, Michael J. Mills, David A. Bailey, Andrew P. Barrett

**Affiliations:** ^1^ Department of Atmospheric and Oceanic Sciences, University of Colorado Boulder, Boulder, CO, USA.; ^2^ Institute of Arctic and Alpine Research, University of Colorado Boulder, Boulder, CO, USA.; ^3^ Climate and Global Dynamics Laboratory, National Center for Atmospheric Research, Boulder, CO, USA.; ^4^ Cooperative Institute for Research in Environmental Sciences, University of Colorado Boulder, Boulder, CO, USA.; ^5^ Atmospheric Chemistry Observations and Modeling Laboratory, National Center for Atmospheric Research, Boulder, CO, USA.; ^6^ National Snow and Ice Data Center, University of Colorado Boulder, Boulder, CO, USA.

## Abstract

The mechanisms underlying decadal variability in Arctic sea ice remain actively debated. Here, we show that variability in boreal biomass burning (BB) emissions strongly influences simulated Arctic sea ice on multidecadal time scales. In particular, we find that a strong acceleration in sea ice decline in the early 21st century in the Community Earth System Model version 2 (CESM2) is related to increased variability in prescribed BB emissions in the sixth phase of the Coupled Model Intercomparison Project (CMIP6) through summertime aerosol-cloud interactions. Furthermore, we find that more than half of the reported improvement in sea ice sensitivity to CO_2_ emissions and global warming from CMIP5 to CMIP6 can be attributed to the increased BB variability, at least in the CESM. These results highlight a new kind of uncertainty that needs to be considered when incorporating new observational data into model forcing while also raising questions about the role of BB emissions on the observed Arctic sea ice loss.

## Teaser

The sensitivity of sea ice to fire emissions highlights climate model uncertainty related to the accuracy of prescribed forcings.

## INTRODUCTION

Arctic sea ice has experienced marked reductions in extent, thickness, and volume in recent decades, making it one of the most striking manifestations of anthropogenic climate change. Sea ice loss has been observed in all months of the year (*1*), but particularly notable is the loss of late-summer sea ice, with reductions in September ice extent and thickness since 1979 of roughly 45 and 66%, respectively (*1*, *2*). However, this loss has not occurred at the same rate year after year. September sea ice loss was largest in the early 21st century, reaching −13.3% per decade over the 14-year period of 1993–2006 (*3*), but the next 14 years have seen a slowdown of the rate of sea ice decline (*4*), with the 2007–2020 sea ice loss trend decreasing to −4.0% per decade (*3*). It is possible that these changes in sea ice loss rate are due solely to internal climate variability; it is well established that internal variability can lead to periods of up to two decades of enhanced or negligible Arctic sea ice loss even as global temperatures rise (*5*–*7*). However, it is also possible that there is a previously unidentified forced contribution to the observed change in sea ice loss trends. This could help explain why climate models are largely not able to simulate the observed rate of sea ice loss without also simulating stronger global warming than observed (*8*–*10*).

Recent work has shown that the Arctic in particular is very sensitive to forcings usually considered less important than anthropogenic greenhouse gas changes. For instance, a modeling study showed that without increases in industrial aerosol emissions since 1920, the Arctic would not have experienced any 50-year cooling trends over the past century (*11*). The subsequent reductions in anthropogenic aerosols emissions since the 1980s, in turn, may have warmed the Arctic surface (*12*–*14*). Emissions of ozone-depleting substances have also been shown to enhance Arctic warming and sea ice loss in the second half of the 20th century (*15*). Furthermore, recent work suggests that biomass burning (BB) emissions from forest fires, which mostly consist of primary organic aerosols, black carbon, and reactive gases, have the potential to change the Arctic aerosol population and affect the rate of sea ice loss (*16*, *17*). This sensitivity of Arctic sea ice to BB aerosols is highly concerning given the severe wildfire seasons that have occurred in recent years (*18*–*20*). On the other hand, increasing large wildfires during autumn over the western United States have been shown to be fueled by more fire-favorable weather associated with declines in Arctic sea ice during preceding months (*21*), highlighting the complex interactions between fires and Arctic climate change and the challenges this poses for state-of-the-art climate models, which do not interactively simulate forest fires but instead use prescribed BB forcing.

Our analysis reveals that a large increase in the interannual variability of prescribed BB emissions from wildfires from 1997 to 2014 in the sixth phase of the Climate Model Intercomparison Project (CMIP6) historical simulations (*22*) affects the multidecadal trends in Arctic sea ice in the Community Earth System Model version 2 (CESM2) (*23*). The abrupt increase in variability in the prescribed BB emissions for CMIP6 is due to a change in available observed BB emission data, rather than reflecting an actual sudden increase in BB emission variability. In CMIP6, satellite-based emissions from the Global Fire Emissions Database (GFED) version 4 with small fires (*24*) from 1997 to 2014 were combined with either proxy records (when available) or fire models to estimate historical BB emissions starting in 1750 (*22*). By comparison, in the previous phase of CMIP (CMIP5), decadal means were used to construct the historical gridded BB emissions (*25*) such that the change in variability in the source datasets at the start of the GFED era did not affect the variability of prescribed BB emissions. As neither the decadally averaged emissions nor the abrupt increase in BB variability is realistic, the resulting uncertainty introduced into the simulated Arctic sea ice loss is due to forcing uncertainty (*26*). This source of uncertainty is often overlooked but needs to be considered when interpreting climate model simulations, in addition to the established uncertainties related to model structure, internal variability, and, for future simulations, emissions scenario (*27*, *28*).

In this study, we show that the increased interannual variability in prescribed CMIP6 historical BB emissions starting in 1997 leads to an acceleration of simulated early 21st century Arctic sea ice loss in the CESM2 Large Ensemble (CESM2-LE) (*29*) due to nonlinear aerosol-cloud interactions during the melt season. We identify this link by performing sensitivity experiments in which we remove the increased BB variability from the CMIP6 historical forcing while conserving the total integrated amount of BB emissions from 1997 to 2014. To isolate forced contributions to the Arctic sea ice evolution, we primarily focus on ensemble means, which reflect the model response to external forcing. We further show how this affects simulated sea ice sensitivities in the CESM, before discussing the implications of these model-based findings for the CMIP6 effort and the potential relevance for the observed evolution of Arctic sea ice.

## RESULTS

### Accelerated sea ice loss in CMIP6-forced simulations of the CESM

**Fig. 1.**
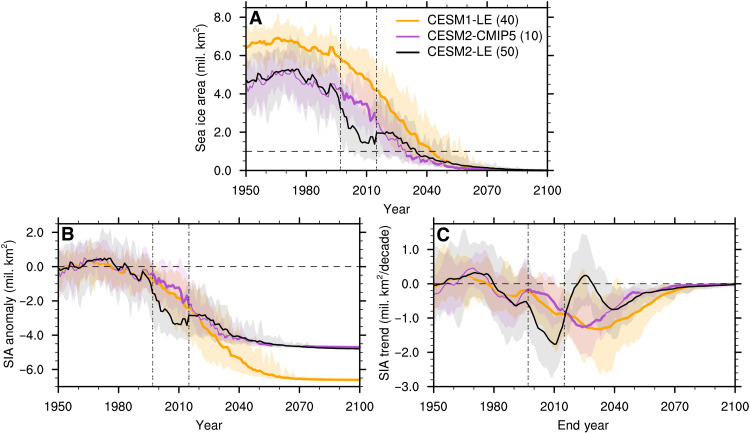
Differences in the rate of Arctic sea ice loss. September (**A**) sea ice area (SIA), (**B**) SIA anomalies relative to the 1940–1969 average, and (**C**) 20-year linear SIA trends in the CESM1-LE, the CESM2-CMIP5, and the CESM2-LE (the ensemble size is indicated in parentheses in the legend). The ensemble mean is shown by the solid line; the full ensemble range is shown by the shading; the horizontal dashed line indicates ice-free conditions in (A), no anomalies in (B), and no trend in (C); and the two vertical double-dashed lines indicate the GFED period. Years when the CESM1-LE and the CESM2-CMIP5 are statistically different from the CESM2-LE at the 95% significance level are indicated with a thicker ensemble mean line and are determined using a two-sample Welch’s *t* test. In (C), values on the *x* axis indicate the end year of the 20-year period over which the linear trend is computed.

Here, we make use of several different CESM ensemble simulations run with different model versions and forcings. These include the CESM1-LE (*30*), a 40-member ensemble of the CESM1 model forced with CMIP5 forcing, the CESM2-CMIP5, a 10-member ensemble of the CESM2 model also forced following the CMIP5 protocol, and the CESM2-LE (*29*), a 50-member ensemble that uses the latest generation of the CESM, the CESM2 (*23*), and is forced using CMIP6 forcing (see Materials and Methods for more details). We find that the evolution of Arctic sea ice area in September throughout the 20th and 21st centuries differs greatly between the two CMIP5-forced versions of the CESM, the CESM1-LE and the CESM2-CMIP5, and the CMIP6-forced version, the CESM2-LE (Fig. 1A). Although the CESM1-LE simulates a much thicker and more extensive sea ice cover compared to both CESM2 experiments before the start of the decline in Arctic sea ice in the later part of the 20th century (*31*), both CMIP5-forced versions of the CESM exhibit a similar rate of Arctic sea ice loss starting in the mid-1990s (Fig. 1, B and C). The CESM1-LE and CESM2-CMIP5 September sea ice area anomaly and trend become gradually more negative with time until the Arctic reaches ice-free conditions every year (*32*). In contrast, the sea ice cover in the CESM2-LE experiences a sharp decline in area starting in the mid-1990s up until the end of the first decade of the 21st century (Fig. 1B), with the ensemble mean sea ice loss trend reaching its highest value of about −1.8 million km^2^/decade around end year 2010 (Fig. 1C). This is followed by a decade-long sea ice recovery in the CESM2-LE ensemble mean until ∼2025 characterized by neutral or even positive trends, after which the ensemble mean area anomaly and trend continue to become more negative until the sea ice cover melts out completely every summer (*31*). Note that this feature of the CESM2-LE sea ice evolution is present regardless of the choice of future CMIP6 emissions scenario (*31*), in all months of the year (fig. S1; although it is most pronounced at the end of the summer), as well as in the version of the CESM2 that uses a high-top atmosphere model, the Whole Atmosphere Community Climate Model version 6 (WACCM6), instead of the standard Community Atmosphere Model version 6 (CAM6) (*31*). The similar rate of Arctic sea ice loss in the CESM1-LE and the CESM2-CMIP5 allows us to conclude that the accelerated sea ice decline in the CESM2-LE is the result of the change in forcing from CMIP5 to CMIP6 and not attributable to differences in model physics between the CESM1 and CESM2 models.

### Impact of BB emissions on simulated Arctic climate

**Fig. 2.**
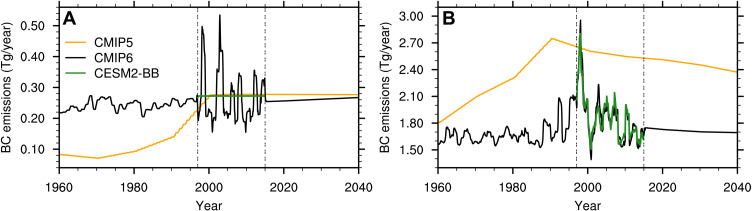
Changes in BB forcing. Prescribed total black carbon (BC) emissions from BB (**A**) from 40° to 70°N and (**B**) globally in CMIP5 (used to force the CESM1-LE and CESM2-CMIP5), CMIP6 (used to force the CESM2-LE), and the CESM2-BB, smoothed with a 12-month running mean. The two vertical double-dashed lines indicate the GFED period. Note that the range of values on the *y* axis is different between the two panels, with higher values of total global black carbon emissions. We use black carbon emissions to represent BB emissions, but all other prescribed BB emissions (dimethyl sulfide, primary organic matter, sulfur dioxide, sulfate aerosols, and secondary organic aerosols) follow a similar time evolution as black carbon (not shown).

We find that the change in prescribed BB emissions from CMIP5 to CMIP6 can explain much of the difference in Arctic sea ice evolution between the CMIP5- and CMIP6-forced CESM simulations (i.e., CESM1-LE and CESM2-CMIP5 versus CESM2-LE). Previous studies suggest that the aerosol forcing of CMIP5 simulations might have been too weak in recent decades (*33*, *34*). In CMIP6, BB emissions were updated to include interannual variability (*22*), rather than using decadal means (*25*) (Fig. 2). Although this decision allows for a more realistic depiction of BB emissions over the recent historical period, it also results in a sudden increase of the interannual variability in BB emissions in 1997 at the start of the GFED era (Fig. 2). This increase in variability is especially pronounced in the Northern Hemisphere (NH) mid-latitudes, where the variability increases by a factor of 5 compared to pre-GFED years (defined here as 1950–1996; Fig. 2A). The interannual variability in global BB emissions increases as well, although only by a factor of 2 (Fig. 2B).

To isolate the impact of the increased BB variability over the GFED era on Arctic sea ice, we conducted sensitivity ensemble simulations (referred to as CESM2-BB hereafter) in which the interannual variability in BB emissions from 1997 to 2014 between 40° and 70°N is removed but the integrated amount of emissions over that same period is retained (Fig. 2A; see Materials and Methods for more details). As a result, the CESM2-BB has prescribed BB emissions over the NH mid-latitudes that are more similar to CMIP5 during the GFED period, with emissions pre- and post-GFED being the same as in CMIP6 (Fig. 2A). Because NH mid-latitude BB emissions make up only ∼14% of the global BB emissions, the variability of global BB emissions is practically unchanged in the CESM2-BB compared to CMIP6 (Fig. 2B).

**Fig. 3.**
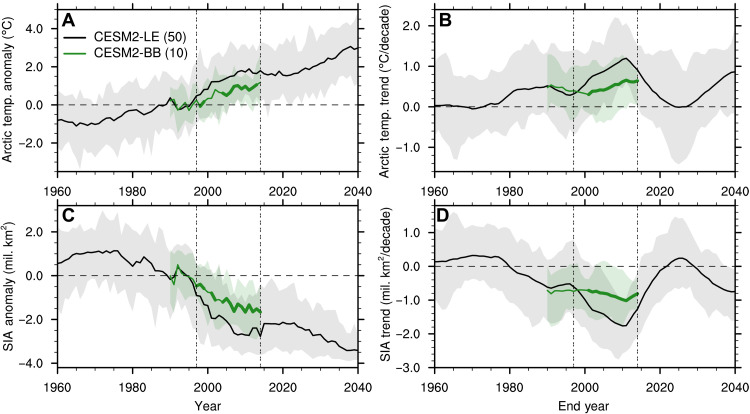
BB emissions impact on Arctic climate. Annual Arctic (70° to 90°N) surface air temperature (**A**) anomalies relative to the 1990–1996 average (when the two simulations share the same forcing) and (**B**) 20-year linear trends, and September sea ice area (SIA) (**C**) anomalies relative to the 1990–1996 average and (**D**) 20-year linear trends in the CESM2-LE and the CESM2-BB (the ensemble size is indicated in parentheses in the legend). The ensemble mean is shown by the solid line, the full ensemble range is shown by the shading, the horizontal dashed line indicates no anomalies in (A) and (C) and no trend in (B) and (D), and the two vertical double-dashed lines indicate the GFED period. Years when the CESM2-BB is statistically different from the CESM2-LE at the 95% significance level are indicated with a thicker CESM2-BB ensemble mean line and are determined using a two-sample Welch’s *t* test. Note that while the CESM2-BB has a smaller ensemble size than the CESM2-LE (10 versus 50 ensemble members), its ensemble size is sufficient to detect a forced sea ice response to the modified BB emissions toward the end of the GFED period (see fig. S2, C and D). In (B) and (D), values on the *x* axis indicate the end year of the 20-year period over which the linear trend is computed.

The sensitivity experiments show that the warming of the Arctic (70° to 90°N) over the GFED period is more pronounced in the CESM2-LE compared to the CESM2-BB (Fig. 3A), with the largest difference over the central and Pacific sectors of the Arctic Ocean (fig. S3). Specifically, the 20-year linear trends in Arctic surface air temperature in the CESM2-LE are significantly larger than the CESM2-BB over most of the GFED period (Fig. 3B), after which the trends reduce to neutral values in the ensemble mean around end year 2025. In addition, the September Arctic sea ice area anomaly and trends are reduced (i.e., less negative) in the CESM2-BB compared to the CESM2-LE over the GFED period (Fig. 3, C and D). Similar results are found not just at the sea ice minimum but in all months of the year, although the difference between the CESM2-BB and the CESM2-LE is most pronounced from July to November (fig. S1). This reduction in the rate of Arctic sea ice decline over the GFED era in the CESM2-BB is not limited to a specific region but is present everywhere in the central Arctic Ocean and particularly over the Pacific sector of the Arctic (fig. S4). Note that this holds true even when looking at five different 10-member subsets of the CESM2-LE to account for the difference in ensemble size with the CESM2-BB. As only the interannual variability in BB emissions over the GFED period differs between the two ensembles, these results allow us to conclude that the increased BB variability in CMIP6 over the GFED period is causing enhanced Arctic warming and sea ice decline in the late 1990s and early 2000s in the CESM2-LE. Note that the impact of the increased variability of BB emissions is not only limited to the Arctic but is also present north of 30°N, as shown in a companion paper that uses the same sensitivity simulations (*35*).

Around year 2010, the trend in Arctic warming and sea ice decline starts to lessen in the CESM2-LE (Fig. 3, B and D), slightly before the start of the future scenario with no BB variability (Fig. 2). This plateau in the temperature and sea ice response is also present in our sensitivity runs with smoothed BB emissions, although to a lesser extent. This leads us to believe that, while the reduced variability in BB emissions in the later part of the GFED period compared to the earlier part of GFED may play a role in contributing to this slowdown in Arctic warming and sea ice decline (Fig. 2), a different forcing or combination of forcings is likely also at play here and should be investigated in the future.

**Fig. 4.**
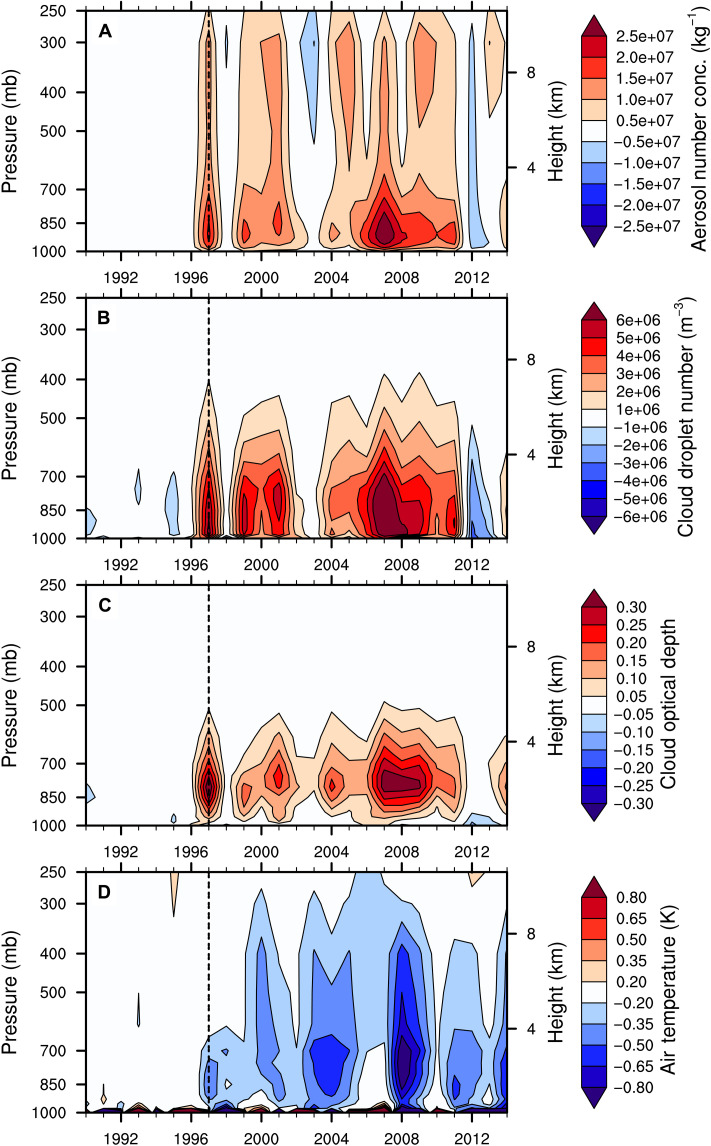
BB emissions impact on Arctic aerosol-cloud interactions. Difference (CESM2-BB − CESM2-LE) in Arctic (70° to 90°N) summer (June-July-August) (**A**) number concentration of aerosols in the accumulation mode, (**B**) cloud droplet number concentration, (**C**) cloud optical depth, and (**D**) air temperature with height. Positive differences (red) indicate larger values in the CESM2-BB, and negative differences (blue) indicate larger values in the CESM2-LE. The vertical double-dashed line indicates the start of the GFED period.

The impact of BB emissions on Arctic climate can be explained by aerosol-cloud interactions (Fig. 4). Freshly emitted BB particles are specified to be hydrophobic (primary carbon mode) in the CESM model and hence cannot initially serve as cloud condensation nuclei (CCN). Through microphysical aging processes, these BB particles gradually become hydrophilic (*36*, *37*). We find that the interannual variability in BB emissions over the NH mid-latitudes in the CESM2-LE (Fig. 2A) is reflected in the Arctic summertime number concentration of aerosols in the primary carbon mode (fig. S5A), showing that fresh BB aerosols from those emissions are transported to the Arctic. However, the signature of the interannual variability in BB emissions is partly lost for the aged aerosols (i.e., those that can act as CCN; Fig. 4A). Specifically, years with smaller BB emissions in the CESM2-LE compared to the CESM2-BB (i.e., 1997, 1999–2001, and 2004–2011; see Fig. 2A) result in lower Arctic summertime number concentration of aerosols in the accumulation mode. The larger aerosol emissions in the CESM2-BB during those years lead to larger aerosol numbers with smaller aerosol diameter compared to the CESM2-LE (Fig. 4A). However, the opposite is not true for years with larger BB emissions in the CESM2-LE than in the CESM2-BB (i.e., 1998, 2002–2003, and 2012–2014; see Fig. 2A). During those years, there is very little difference between the two CESM simulations in terms of aerosol number concentration (Fig. 4A). This asymmetric response is likely a reflection of the observed nonlinear and saturated response of CCN to aerosol loading (*38*, *39*), as it has been previously shown that cloud albedo has a nonlinear response to aerosol emissions that diminishes with increasing emissions [(*39*), see their figure 3]. As a result of the larger concentration of summertime aerosols in the accumulation mode in the CESM2-BB in years with larger NH mid-latitude BB emissions, we find larger cloud droplet number concentration in the CESM2-BB compared to the CESM2-LE, especially close to the surface and up to about 500 mb (Fig. 4B). This results in higher lower-tropospheric cloud optical depth compared to the CESM2-LE over the GFED period (Fig. 4C) through indirect aerosol-cloud interactions, specifically the Twomey effect (*40*). The higher cloud optical depth is associated primarily with increases in cloud liquid amount (fig. S5B) and leads to a net cooling from the surface up to about 300 mb (Fig. 4D). Although the local impact of an increased aerosol loading in the Arctic is the nonlinear result of competing cooling and warming aerosol indirect effects (*17*), the decrease in Arctic surface reflectivity during the melt season shifts the aerosol indirect effect toward cooling (*41*). Note that the temperature response toward the end of the GFED period is likely enhanced through snow/ice albedo feedback as the extent of the sea ice cover starts to significantly differ between the two ensembles (Fig. 3C).

### Impact of BB emissions on sea ice sensitivity

The observed loss of Arctic sea ice has been shown to be tightly coupled to increasing global mean surface air temperature (*42*, *43*) and cumulative anthropogenic CO_2_ emissions (*44*). This metric of sea ice sensitivity to CO_2_ and global warming is commonly used by the sea ice community and has even been proposed as a way to reduce the uncertainty range of future sea ice evolution (*44*, *45*). Previous literature has shown that models usually simulate a lower sensitivity of Arctic sea ice loss per degree of global warming than has been observed (*42*, *44*), with accurate Arctic sea ice retreat only in CMIP5 runs that have too much global warming, which suggests that models may be getting the right Arctic sea ice retreat for the wrong reasons (*10*). More recently, the CMIP6 multimodel ensemble mean was shown to provide a more realistic estimate of the sensitivity of September Arctic sea ice area to a given amount of anthropogenic CO_2_ emissions and global warming compared with earlier CMIP experiments (*9*). It was, however, unclear whether this change reflects an improvement of model physics or primarily arises from differences in the historical forcing in CMIP6 relative to CMIP5, in particular, differences in BB emissions and ozone (*9*).

**Fig. 5.**
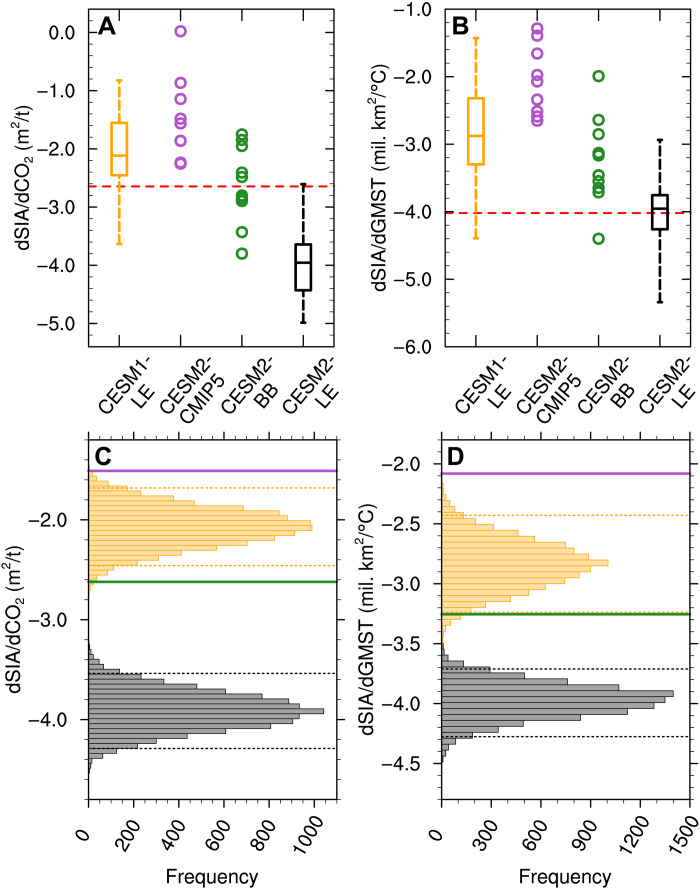
BB emissions impact on sea ice sensitivity. Sea ice sensitivity to (**A**) cumulative anthropogenic CO_2_ emissions (defined as the change in Arctic September sea ice area per change in cumulative anthropogenic CO_2_ emissions in m^2^ per metric ton of CO_2_) and (**B**) global annual mean surface temperature (defined as the change in Arctic September sea ice area per change in global mean surface temperature in million km^2^ per °C) from 1979 to 2014 in the CESM1-LE, the CESM2-CMIP5, the CESM2-BB, and the CESM2-LE, with the red dashed line showing the observed sensitivity. For the two large ensembles, the box shows the interquartile range, the line inside the box shows the median, and the whiskers show the minimum and maximum across all ensemble members. For the CESM2-CMIP5 and the CESM2-BB, the circles indicate the sea ice sensitivity of the 10 ensemble members. Histograms of sea ice sensitivity to (**C**) cumulative anthropogenic CO_2_ emissions and (**D**) global annual mean surface air temperature obtained by bootstrapping the CESM1-LE and CESM2-LE ensemble means with 10 members 10,000 times with replacement, with the dotted lines showing the 95% confidence range for each distribution. The color scheme for the histograms is the same as in (A) and (B), and the purple and green lines indicate the ensemble mean sensitivity of the CESM2-CMIP5 and the CESM2-BB, respectively.

In agreement with what was reported for CMIP6 models as a group (*9*), we find that the sea ice sensitivity to cumulative anthropogenic CO_2_ emissions and global mean surface temperature is generally higher in the CMIP6-forced version of the CESM, the CESM2-LE, compared to the two CMIP5-forced versions, the CESM1-LE and the CESM2-CMIP5 (Fig. 5, A and B). In contrast, the sea ice sensitivity of the CESM2-BB falls somewhere in between the range of sea ice sensitivities of the CMIP5-forced versions of the CESM and the CESM2-LE, although all 10 ensemble members of the CESM2-BB overlap with at least one of the large ensemble distributions, if not both. Note that trends in September sea ice area and global mean surface temperature are related in these simulations, with more sea ice loss present in simulations with more global warming. Hence, the change in sea ice sensitivity to global mean surface temperature in the CESM2-BB is influenced by both factors. Using bootstrapping, we show that the sea ice sensitivity of the CESM2-BB ensemble is statistically distinct from the CESM1-LE and the CESM2-LE at the 95% confidence level when accounting for the smaller ensemble size of the CESM2-BB (Fig. 5, C and D). Note that bootstrapping, or randomly resampling with replacement to generate statistics, requires no distribution assumptions and is only possible with sufficiently large ensembles. By comparing the means of the two bootstrapped distributions, we are able to attribute about 70 and 64% of the increased sea ice sensitivity to CO_2_ and global warming, respectively, from the CESM1-LE to the CESM2-LE to the enhanced variability in BB emissions. When looking at the increase in sea ice sensitivity from CMIP5 to CMIP6 only within the CESM2, the part that can be attributed to the increased BB variability drop slightly to 54 and 39%, although our confidence in these numbers is lower due to the smaller ensemble size of the CESM2-CMIP5 and the large variability across ensemble members. Hence, the enhanced variability in BB emissions from CMIP5 to CMIP6 in the CESM seems to be responsible for more than half of the increased sea ice sensitivity to CO_2_ and global warming recently reported by the Sea-Ice Model Intercomparison Project (SIMIP) Community for CMIP6 in general (*9*), with the rest related to other changes in historical forcing and/or improvement of model physics. This is especially true for the sea ice sensitivity to CO_2_, as temperature is also affected by the change in BB emissions but CO_2_ concentrations are typically prescribed in CMIP6 simulations.

## DISCUSSION

We showed that a large part of the enhanced early 21st century Arctic surface warming and September sea ice decline in the CESM2-LE compared to the CESM1-LE and the CESM2-CMIP5 can be attributed to the increased interannual variability in prescribed NH mid-latitude BB emissions in the CMIP6 forcing compared to CMIP5. Specifically, we showed that the increased BB variability results in surface warming due to nonlinear aerosol-cloud interactions, as decreased cloud optical depth during years with low BB-related aerosol burdens enhances warming more than years with high BB-related aerosol burdens lead to cooling. Hence, the increased BB variability over the GFED period leads to an additional forced sea ice loss in the CESM2-LE beyond the one driven by increases in greenhouse gases (*46*) and internal variability (*5*, *47*, *48*). The presence of this non-greenhouse gas-forced simulated sea ice loss in the early 21st century also affects the sea ice sensitivity, a metric often used to evaluate model performance (*9*, *32*, *43*, *44*). Specifically, we find that the increased interannual variability in BB emissions during the GFED era explains over half of the increase in sea ice sensitivity to CO_2_ emissions and global warming from the CMIP5-forced to the CMIP6-forced versions of the CESM. This is the second time that aerosol-related forcing changes have been shown to affect Arctic sea ice trends between CMIP generations (*49*), highlighting how sensitive sea ice is to the effects of aerosol emissions. The sensitivity of the CESM2 to changes in BB variability also raises the question as to whether the lack of interannual variability in aerosol forcing in the preindustrial control and future scenario runs could be problematic.

It is not only the CESM2 that shows an increase of the rate of Arctic sea ice decline over the GFED period, but some other CMIP6 models do as well (figs. S6 and S7). From the 12 additional CMIP6 models assessed here (see Materials and Methods), 4 (i.e., ACCESS-ESM1.5, FGOALS-g3, MIROC6, and MPI-ESM1.2-HR) show an accelerated ensemble mean sea ice loss over the GFED period, although none of them is as large as the CESM2. This indicates that the impact of BB emissions is likely not just limited to the CESM2 but may affect other CMIP6 models as well, in agreement with results from a companion paper that finds increased surface downwelling shortwave radiation during the GFED period in several CMIP6 models in addition to the CESM2 (*35*). Furthermore, the fact that some CMIP6 models show a similar sea ice loss acceleration as the one attributed to the new BB emissions in the CESM2, while others do not, calls for a better understanding of intermodel differences in light of their sensitivity to aerosol emissions. In particular, the details of the cloud microphysics scheme used to represent aerosol-cloud interactions may be responsible for the degree to which a model responds to the BB forcing. It was recently shown that removing an inappropriate limiter on cloud ice number in the CESM2 and decreasing the time-step size can result in 20% smaller aerosol-cloud interaction (*50*). This could help explain why the impact of the BB variability is larger in the CESM2 compared to the other CMIP6 models assessed here.

Overall, our analysis shows that BB emissions can influence multidecadal variations in Arctic sea ice. This work also demonstrates that changes in the variability of emissions, not just changes in the mean, can have large effects on climate through nonlinear cloud feedbacks (*51*). Hence, our findings suggest that the way short-lived climate forcings like BB emissions are prescribed in models can have unexpected remote effects in vulnerable regions such as the Arctic. This highlights the challenges associated with incorporating newly available observations into climate forcing datasets and demonstrates the impact of forcing uncertainty that arises from imperfect knowledge or representation of climate forcings in model simulations (*26*). To reduce the forcing uncertainty related to BB emissions, which requires avoiding a sharp increase in BB variability in 1997 while still making use of the new satellite-based observations over the GFED period, we recommend reassessing the variability of emissions pre-GFED, potentially through the use of an interactive fire model. Similarly, interannual variability in BB emissions could be introduced into future scenarios by coupling fire-enabled dynamic global vegetation models with climate and atmospheric chemistry models, which allows for feedbacks between fire and climate to be simulated (*52*, *53*). The Fire Model Intercomparison Project (FireMIP) is actively working on developing modeling capacity to predict the trajectory of fire-regime changes in response to projected future climate and land-use changes (*54*).

**Fig. 6.**
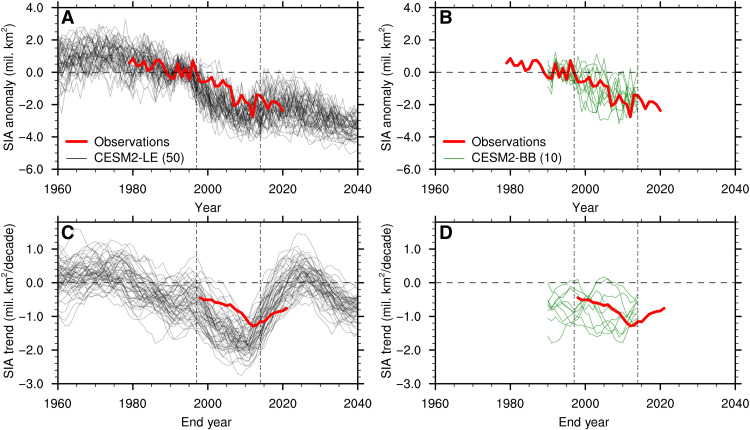
Potential impact of BB emissions on observed Arctic sea ice decline. September sea ice area (SIA) (**A** and **B**) anomalies relative to the 1990–1996 average (when the two simulations share the same forcing) and (**C** and **D**) 20-year linear trends in each individual ensemble member of the (A and C) CESM2-LE and the (B and D) CESM2-BB (the ensemble size is indicated in parentheses in the legend) compared to observations. The horizontal dashed line indicates no anomalies in (A) and (B) and no trend in (C) and (D), and the two vertical double-dashed lines indicate the GFED period. In (C) and (D), values on the *x* axis indicate the end year of the 20-year period over which the linear trend is computed.

Last, the early GFED period stands out as particularly variable in BB emissions north of 40°N, both in the real world and in the CMIP6 forcing (*22*). As discussed earlier, several studies have documented a steepening of the observed trend of Arctic sea ice decline since the mid-1990s (*55*, *56*) and a smaller trend since 2007 (*3*, *7*). This qualitatively matches the behavior simulated by almost all 50 ensemble members of the CESM2-LE (Fig. 6C) and some other CMIP6 models (fig. S7). In contrast, only a few ensemble members of the CESM2-BB simulate a similar increase in negative sea ice area trend over the GFED period as seen in the observations (Fig. 6D). This raises the question of a potential role of BB emissions in the observed Arctic sea ice loss since the late 1990s. On the other hand, this is challenging to diagnose given the limitations of pre-GFED BB emission observations and the substantial role of internal variability on Arctic sea ice (*5*, *6*, *7*, *48*, *57*). The large impact of internal variability on sea ice anomalies in an individual realization is clearly visible in both CESM2-LE and CESM2-BB simulations (Fig. 6, A and B). Nonetheless, our results indicate that BB emission variability strongly influences simulated multidecadal Arctic sea ice trends in the CESM2-LE. Hence, the potential impact of the variability of BB emissions on the observed Arctic sea ice loss should be further investigated. This is especially timely given the record Arctic fire years in 2019 and 2020 (*18*, *19*, *20*), the recent observed positive trend in burned area and severity of NH wildfires (*58*, *59*, *60*), and the projected increase in wildfires in the future (*61*, *62*).

## MATERIALS AND METHODS

### Observational data

Observed estimates of NH monthly sea ice area since the beginning of the continuous satellite record in 1979 are from the National Snow and Ice Data Center (NSIDC) Sea Ice Index version 3 (*63*), with the observational pole hole filled assuming sea ice concentration of 100%. Historical anthropogenic CO_2_ emissions are taken from the historical budget of the Global Carbon Project (*64*). For global mean surface temperature, we use estimates from GISTemp v4 (*65*, *66*) and calculate anomalies relative to the period 1850–1900.

### CESM simulations

The CESM1-LE (*30*) is a 40-member ensemble of the CESM1.1 model (*67*) that has been widely used for Arctic sea ice studies and generally performs well when compared to observations (*47*, *68*, *69*, *70*). Historical simulations span 1920 to 2005, while the RCP8.5 scenario simulations cover 2006 to 2100. The CESM1-LE uses the Community Atmosphere Model version 5 (CAM5) (*67*) along with a three-mode version of the Modal Aerosol Module (MAM3) (*71*), and cloud-aerosol interactions are represented by the MG1 cloud microphysics scheme (*72*).

With several science and infrastructure improvements, the CESM2 model (*23*) is the latest generation of the CESM and NCAR’s (National Center for Atmospheric Research) contribution to CMIP6. Specifically, aerosols are simulated through the use of the MAM4 approach (*73*), and cloud-aerosol interactions are represented by the updated Morrison and Gettelman scheme (MG2) (*74*). The CAM5 shallow convection, planetary boundary layer, and cloud macrophysics schemes are replaced in CESM2 with a unified turbulence scheme, the Cloud Layers Unified By Binormals (CLUBB) (*75*). As a result of these improvements, the CESM2 shows large reductions in low-latitude precipitation and short-wave cloud radiative forcing biases, leading to improved historical simulations with respect to available observations compared to its previous major release, the CESM1.1 used in the CESM1-LE (*23*). Two separate CESM2 configurations have been contributed to the CMIP6 effort, differing only in their atmosphere component: the “low-top” (40 km, with limited chemistry) CAM6 (referred to as CESM2) (*23*) and the “high-top” (140 km, with interactive chemistry) WACCM6 (referred to as CESM2-WACCM) (*76*). Previous analysis has shown that the low-top CESM2 simulates a thinner 20th century sea ice cover than the high-top CESM2-WACCM (*77*) and the CESM1-LE (*31*). Most of the analysis presented here focuses on a recently released large initial-condition ensemble (referred to as CESM2-LE) that uses the version of the CESM2 with CAM6 as the atmosphere component (*29*), but results from the CESM2-WACCM (*78*, *79*) are also included in the comparison with other CMIP6 models (figs. S6 and S7).

The CESM2-LE (*29*) is a 100-member large ensemble suite that was run from 1850 to 2014 under historical forcing and from 2015 to 2100 following the medium-to-high SSP3-7.0 scenario (*80*). The CESM2-LE initialization procedure was designed to include a mix of macro- and micro-perturbations, where macroperturbations were initialized from 20 independent restart files at 10-year intervals (total of 20 ensemble members) and microinitializations involved a small random perturbation in 20 members for four different start years of the preindustrial control simulation meant to represent different AMOC (Atlantic Meridional Overturning Circulation) states (total of 80 ensemble members). Note that most of this study focuses on the first 50 members of the CESM2-LE (referred to as CESM2-LE) since those follow CMIP6 protocols in terms of BB emissions (*22*). For the second set of 50 members [referred to as BB_CMIP6_SM, as in the CESM2-LE overview paper (*29*)], the CMIP6 global BB emissions of all relevant species were smoothed in time from 1990 to 2020 to remove interannual variability based on the climate impacts of the high BB variability over the GFED period, as presented in this paper and a companion paper (*35*). Note that the code base for the BB_CMIP6_SM also incorporates corrections for two sets of errors that were present in the CESM2-LE: (i) error in the SO_2_, SO_4_, and gas-phase semivolatile secondary organic aerosol emission datasets and (ii) the presence of sporadic large CO_2_ uptake over land (*29*). These minor corrections did not result in any pronounced climate-changing impacts relative to the CESM2-LE.

To isolate the impact of the change in model version from CESM1 to CESM2 versus the change in forcing from CMIP5 to CMIP6, we also make use of a new set of transient simulations with CESM2 under CMIP5 forcing. The forcing applied in these simulations is consistent with that used in the CESM1-LE. The CESM2-CMIP5 is a 10-member ensemble that was run from 1920 to 2100 and is perfectly suited to disentangle the role of forcing versus structural model changes in the differences between the CESM1-LE and CESM2-LE.

### CESM2 sensitivity experiments with homogenized forcing

To investigate the impact of the increased interannual variability in BB emissions over the GFED period, we ran a set of sensitivity experiments using the CESM2 (referred to as CESM2-BB) in which we averaged BB emissions from 1997 to 2014, computed on a monthly basis, such that BB emissions have a fixed annual cycle while keeping the same integrated amount of emissions over that same period. This approach is identical in nature to what was used in CMIP5 (*25*) and removes any sharp transition with BB emissions over pre-GFED years as well as with the SSP BB emissions since those are homogenized to the averaged GFED emissions. The CESM2-BB simulations are initialized in 1990 from the first 10 members of the CESM2, and only BB emissions over the 40° to 70°N latitudinal band from 1997 to 2014 are modified. This region is chosen to target BB emissions from NH mid-latitude wildfires, but similar results are found by removing the variability in BB emissions globally instead of only between 40° and 70°N (not shown), which highlights the impact of NH mid-latitude fires on Arctic climate. These sensitivity simulations are the same as the first 10 ensemble members used in a companion paper (*35*).

Although the ensemble size of the CESM2-BB is much smaller compared to the CESM2-LE, we find that 10 ensemble members are enough to detect a forced response to the homogenized BB emissions toward the end of the GFED period in the CESM2. Specifically, we compare the CESM2-LE to the BB_CMIP6_SM (fig. S2, A and B), which also uses homogenized BB emissions to avoid the increase in BB variability over the GFED era (*29*). With 10 ensemble members, we are able to detect a forced response that is statistically different in 2001 and from 2007 to 2011 for the September sea ice area and from 2009 to 2011 and 2025 to 2027 for the 20-year linear trend in September sea ice area (fig. S2, C and D). Note, however, that for the BB_CMIP6_SM, the chosen smoothing technique and years over which the smoothing is applied differ slightly from what we used in the CESM2-BB experiment. In particular, the smoothing in the BB_CMIP6_SM is applied globally over 1990–2020 using an 11-year running mean filter such that the integrated amount of emissions over the GFED period is not the same as in the CMIP6 forcing (or the CESM2-BB). Nonetheless, the Arctic sea ice response to homogenized BB forcing is similar between the BB_CMIP6_SM and the CESM2-BB.

### CMIP6 simulations

We also use simulations from a subset of CMIP6 models that provided at least three ensemble members for the historical and SSP3-7.0 scenario simulations. As of the time of publication of this study, the models that meet this criteria (excluding the CESM2 and CESM2-WACCM described above) are as follows: ACCESS-CM2 (*81*, *82*), ACCESS-ESM1.5 (*83*, *84*), BCC-ESM1 (*85*, *86*), CanESM5 (*87*, *88*), EC-Earth3-Veg (*89*, *90*), FGOALS-g3 (*91*, *92*), IPSL-CM6A-LR (*93*, *94*), MIROC6 (*95*, *96*), MPI-ESM1.2-HR (*97*,*98*), MPI-ESM1.2-LR (*99*, *100*), MRI-ESM2.0 (*101*, *102*), and NorESM2-LM (*103*, *104*). In cases where the ScenarioMIP SSP3-7.0 simulation was not available, we then used the AerChemMIP SSP3-7.0 simulation that uses the same forcing as the ScenarioMIP SSP3-7.0 but only extends to the end of 2055 (*105*). Even if a modeling center provided more than three ensemble members, only the first three are used to allow for a consistent comparison across all CMIP6 models. Although using only CMIP6 models that provide at least three ensemble members limits the total number of CMIP6 models included in our analysis, it is necessary to choose an ensemble size that is large enough to represent the forced sea ice response to BB emissions, as some individual members of the CESM2-LE show different trajectories despite the identified forced response to the BB forcing (Fig. 6A). Using an ensemble size of three members was chosen as a compromise since the ensemble mean of the first three ensemble members of the CESM2-LE matches the full ensemble mean reasonably well while requiring more members would further reduce the number of available CMIP6 models.

### Criteria for determining sensitive versus not sensitive CMIP6 models

The CMIP6 models are separated into a sensitive and not sensitive category based on whether they exhibit a similar sensitivity to the increased variability in BB emissions as the CESM2-LE (figs. S6 and S7). First, we calculate 20-year linear trends in September sea ice area for each model and compare the slope of the 20-year linear trends between the reference period of end years 1978–1990 and the acceleration period of end years 1997–2009. Note that we chose the last year of the acceleration period to be 2009 instead of the last year of the GFED era (i.e., 2014) based on when the CESM2-LE and CESM2-WACCM reach their maximum negative September sea ice area trend (see fig. S7). For a model to be characterized as sensitive, the slope of sea ice area trends over the acceleration period needs to be at least two times larger (in absolute value) than the slope of sea ice area trends over the reference period. This criteria is defined on the basis of the relative increase in sea ice trend for each model to account for the different magnitudes of sea ice loss across all CMIP6 models (fig. S7). We decided to choose two periods of the same length and to exclude the years 1991–1996 from the reference period because of the Mount Pinatubo volcanic eruption in 1991 and the global cooling that followed for a few years, which resulted in a peak increase in Arctic sea ice extent about a year and a half after the eruption in some models (*106*). Note that the classification into the sensitive and not sensitive category is not affected by the choice of reference period or the exact magnitude of the accelerated sea ice loss.
