# Supplementary material for: Enhanced simulated early 21st century Arctic sea ice loss due to CMIP6 biomass burning emissions
Source: Sci Adv. 2022 Jul 27;8(30):eabo2405. doi: 10.1126/sciadv.abo2405 (PMC9328692; doi:10.1126/sciadv.abo2405)
Supplement: Supplementary file 1 — Figs. S1 to S7 [file sciadv.abo2405_sm.pdf]

Science Advances
AAAS

Supplementary Materials for

Enhanced simulated early 21st century Arctic sea ice loss due to CMIP6 biomass burning emissions

Patricia DeRepentigny *et al.*

Corresponding author: Patricia DeRepentigny, patricia.derepentigny@colorado.edu

Sci. Adv. **8**, eabo2405 (2022)
DOI: 10.1126/sciadv.abo2405

This PDF file includes:

Figs. S1 to S7

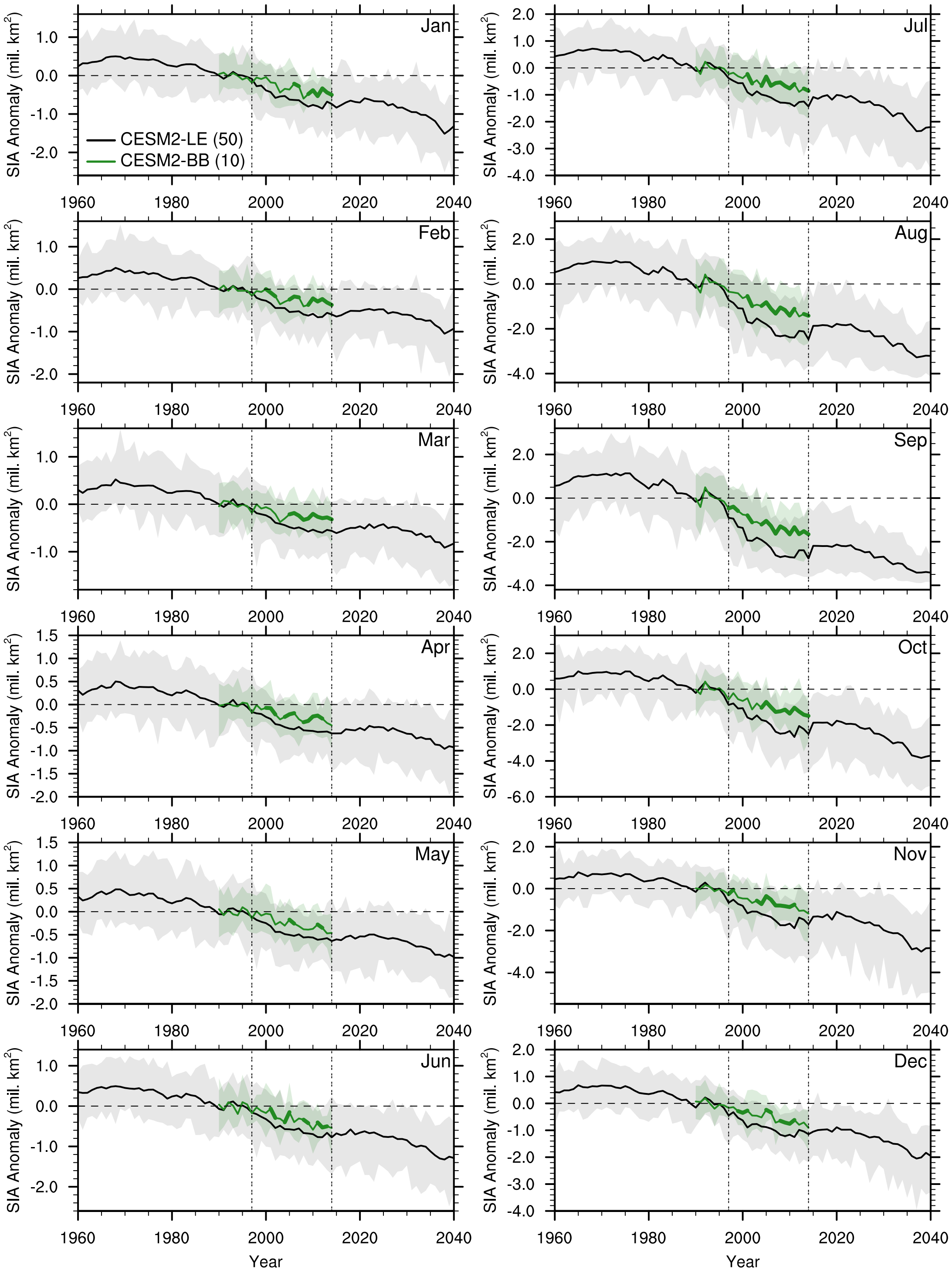

Fig. S1. BB emissions impact on Arctic sea ice in all months. Sea ice area (SIA) anomalies relative to the 1990–1996 average (when the two simulations share the same forcing) in each month of the year in the CESM2-LE and the CESM2-BB. The ensemble mean is shown by the solid line, the full ensemble range is shown by the shading, the horizontal dashed line indicates no anomalies, and the two vertical double-dashed lines indicate the GFED period. Years when the CESM2-BB is statistically different from the CESM2-LE at the 95% significance level are indicated with a thicker CESM2-BB ensemble mean line and are determined using a two-sample Welch's t-test. Note that the range of values on the y-axis varies across all panels.

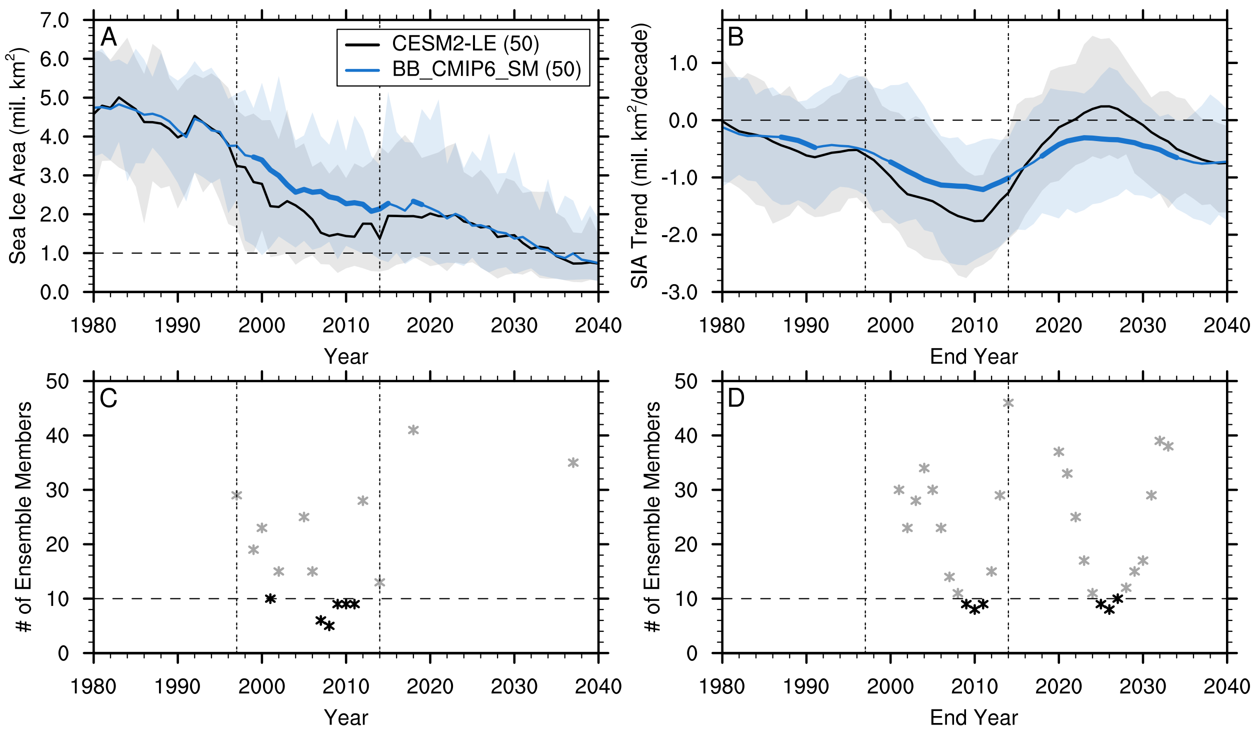

Fig. S2. Minimum number of ensemble members needed to detect a forced response to the homogenized BB emissions. September sea ice area (SIA) (**A**) anomalies relative to the 1940–1969 average and (**B**) 20-year linear trends in the CESM2-LE and the BB_CMIP6_SM (the ensemble size is indicated in parentheses in the legend). The ensemble mean is shown by the solid line and the full ensemble range is shown by the shading. Years when the BB_CMIP6_SM ensemble is statistically different from the CESM2-LE ensemble at the 95% significance level are indicated with a thicker BB_CMIP6_SM ensemble mean line and are determined using a two-sample Welch's t-test. Minimum number of ensemble members needed for the September SIA (**C**) anomalies relative to the 1940–1969 average and (**D**) 20-year linear trends between the CESM2-LE and BB_CMIP6_SM ensembles to be statistically different at the 95% significance level. This is done by bootstrapping the two ensembles 10,000 times with a sub-sample size varying from 2 to 50. Years when 10 ensemble members or less are needed for the two ensembles to be statistically different are highlighted with black stars, while other years are shown with gray stars. The horizontal dashed line indicates ice-free conditions in (A), no trend in (B), and 10 ensemble members in (C and D), and the two vertical double-dashed lines indicate the GFED period. In (B and D), values on the x-axis indicate the end year of the 20-year period over which the linear trend is computed.

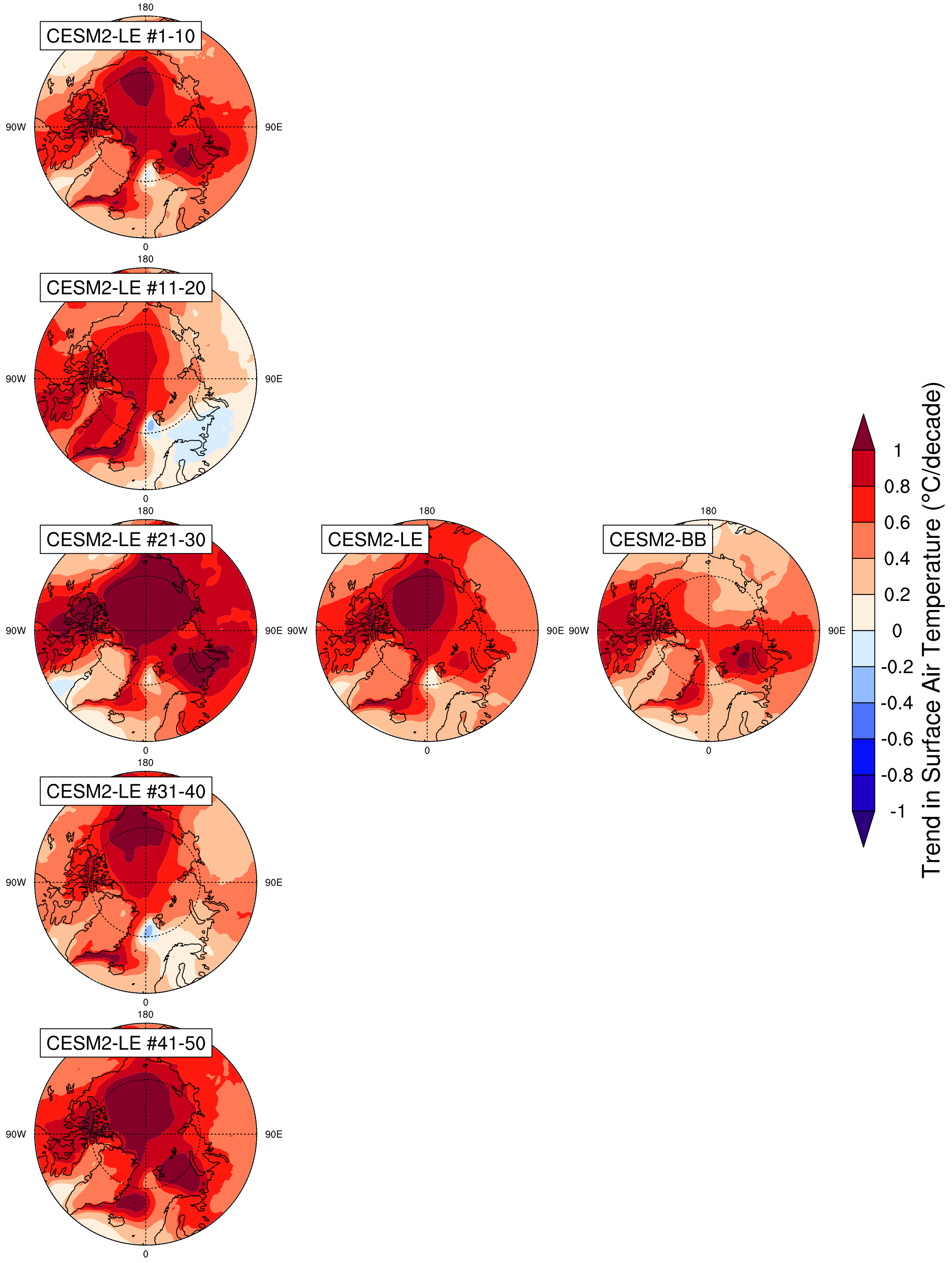

Fig. S3. Spatial patterns of BB impacts on Arctic surface air temperature. Spatial distribution of the linear trend in annual surface air temperature over the GFED period (1997–2014) in five different 10-member ensembles of the CESM2-LE (left), the ensemble mean of the CESM2-LE (middle) and the CESM2-BB (right).

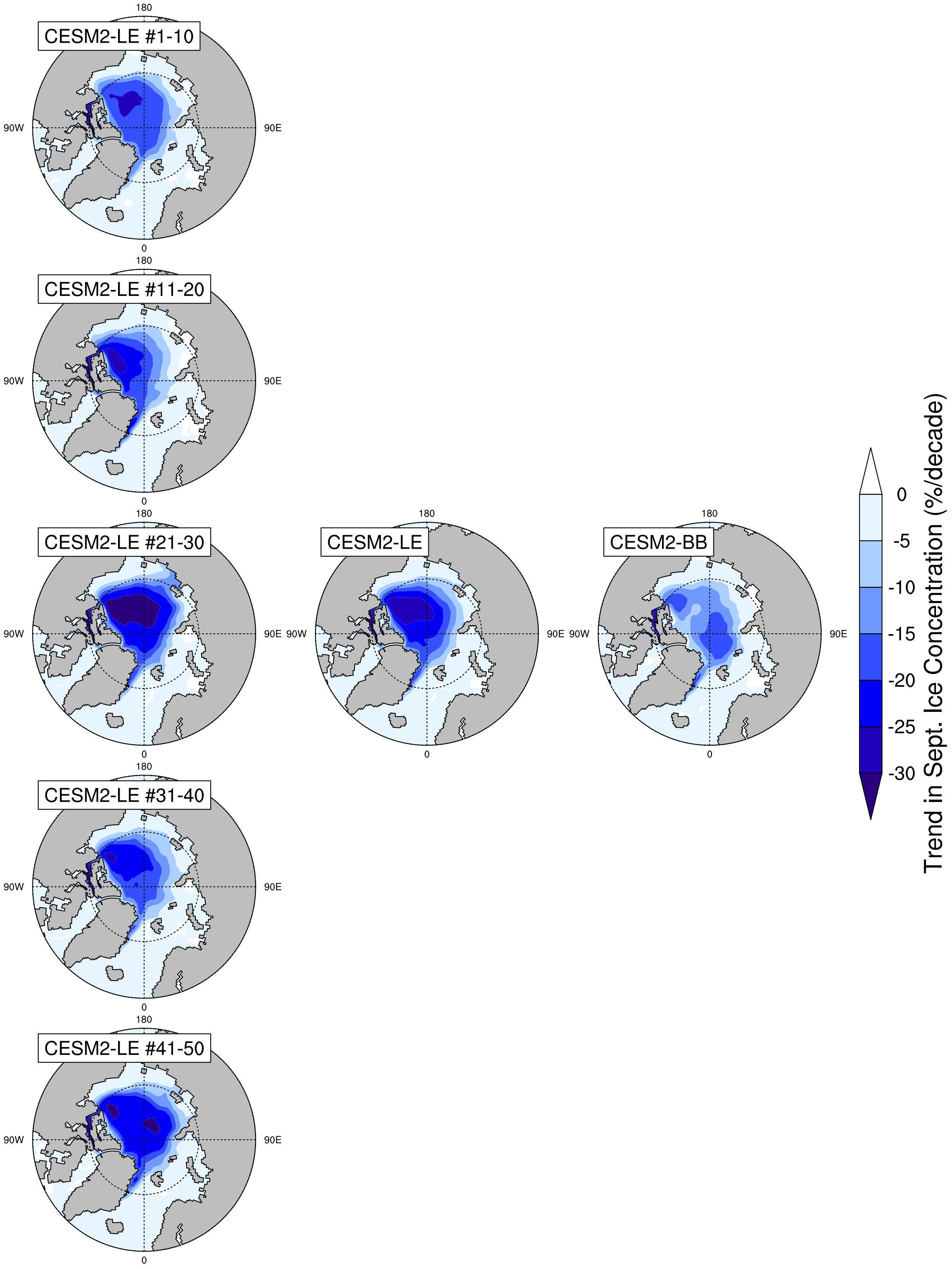

Fig. S4. Spatial patterns of BB impacts on Arctic sea ice concentration. Spatial distribution of the linear trend in September sea ice concentration over the GFED period (1997–2014) in five different 10-member ensembles of the CESM2-LE (left), the ensemble mean of the CESM2-LE (middle) and the CESM2-BB (right).

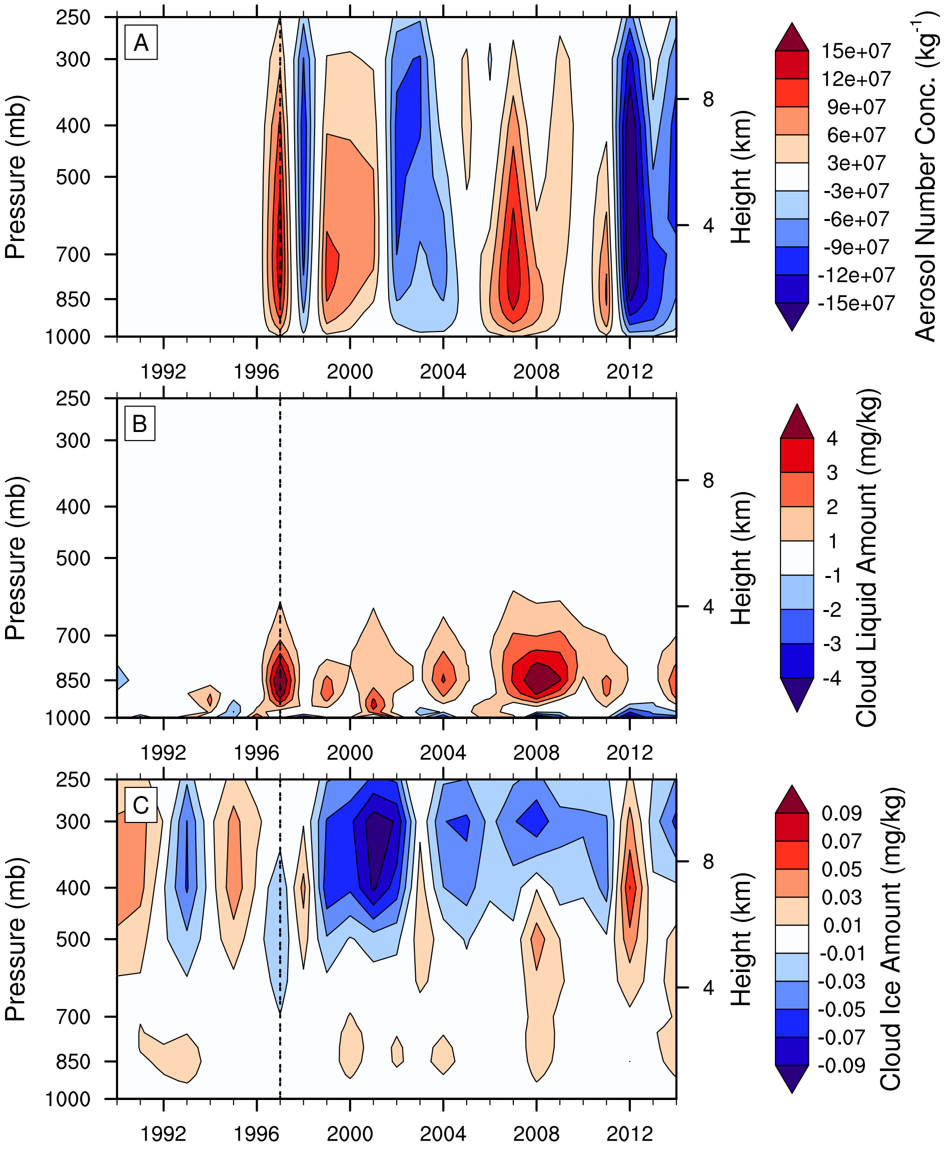

Fig. S5. BB emissions impact on Arctic primary carbon aerosols and clouds. Difference (CESM2-BB – CESM2-LE) in Arctic (70–90°N) summer (JJA) (**A**) number concentration of aerosols in the primary carbon mode as well as cloud (**B**) liquid and (**C**) ice amount with height. Positive differences (red) indicate larger values in the CESM2-BB and negative differences (blue) indicate larger values in the CESM2-LE. The vertical double-dashed line indicates the start of the GFED period. In (B and C), note the same units but different range of the colorbar between the two panels.

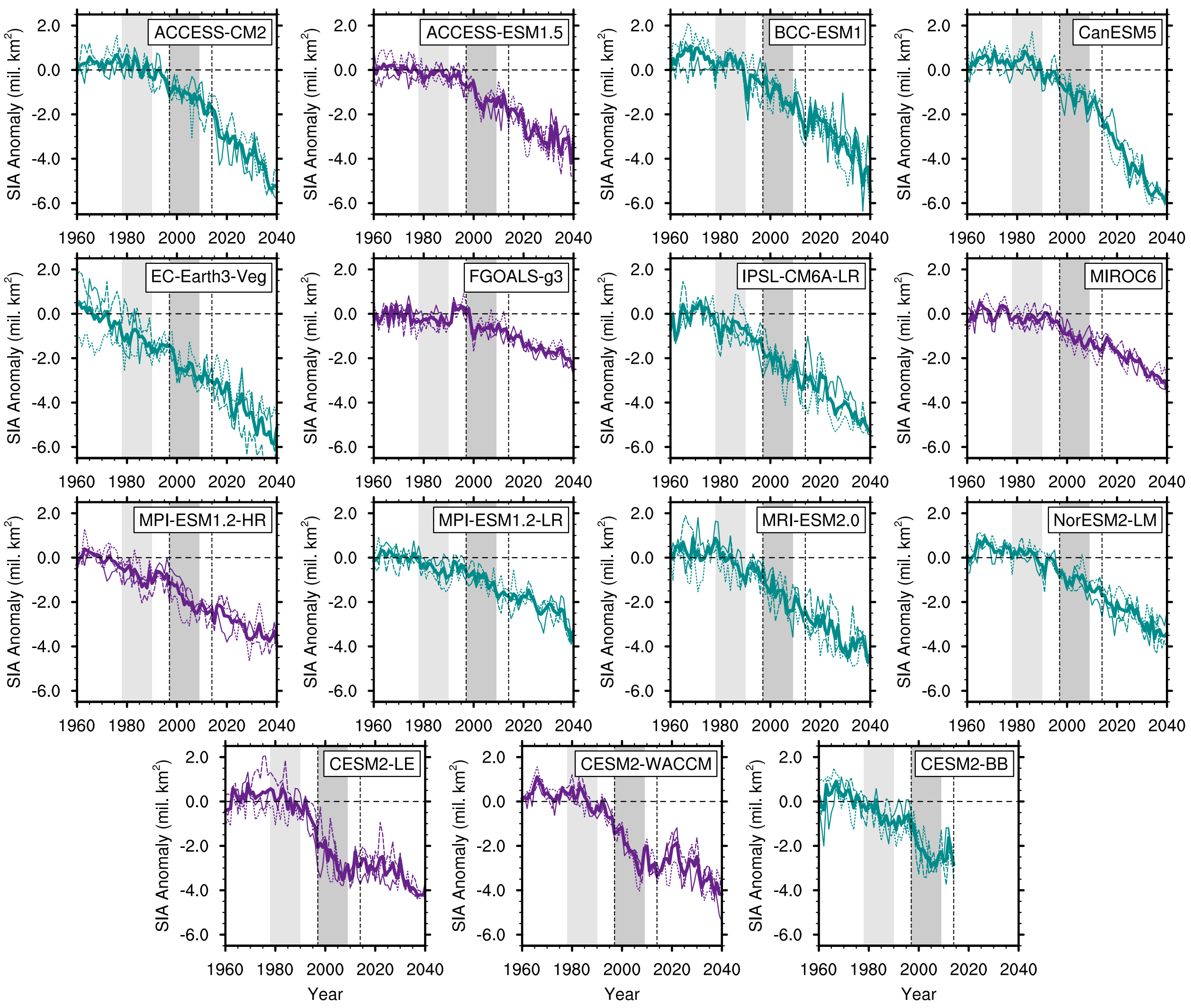

Fig. S6. September sea ice evolution in CMIP6 models. September sea ice area (SIA) anomalies relative to the 1940–1969 average for each CMIP6 model. Models in the sensitive category are shown in purple and the ones in the not sensitive category are shown in turquoise. For each model, the first three ensemble members are shown as thin lines and the ensemble mean is shown by the thick line. The light gray shaded region corresponds to the reference period 1978–1990 and the dark grey shaded region corresponds to the acceleration period 1997–2009 (see Materials and Methods for more details). The horizontal dashed line indicates no anomalies and the two vertical double-dashed lines indicate the GFED period. The last row shows the CESM2-LE, the CESM2-WACCM and the CESM2-BB for comparison, only using the first three ensemble members.

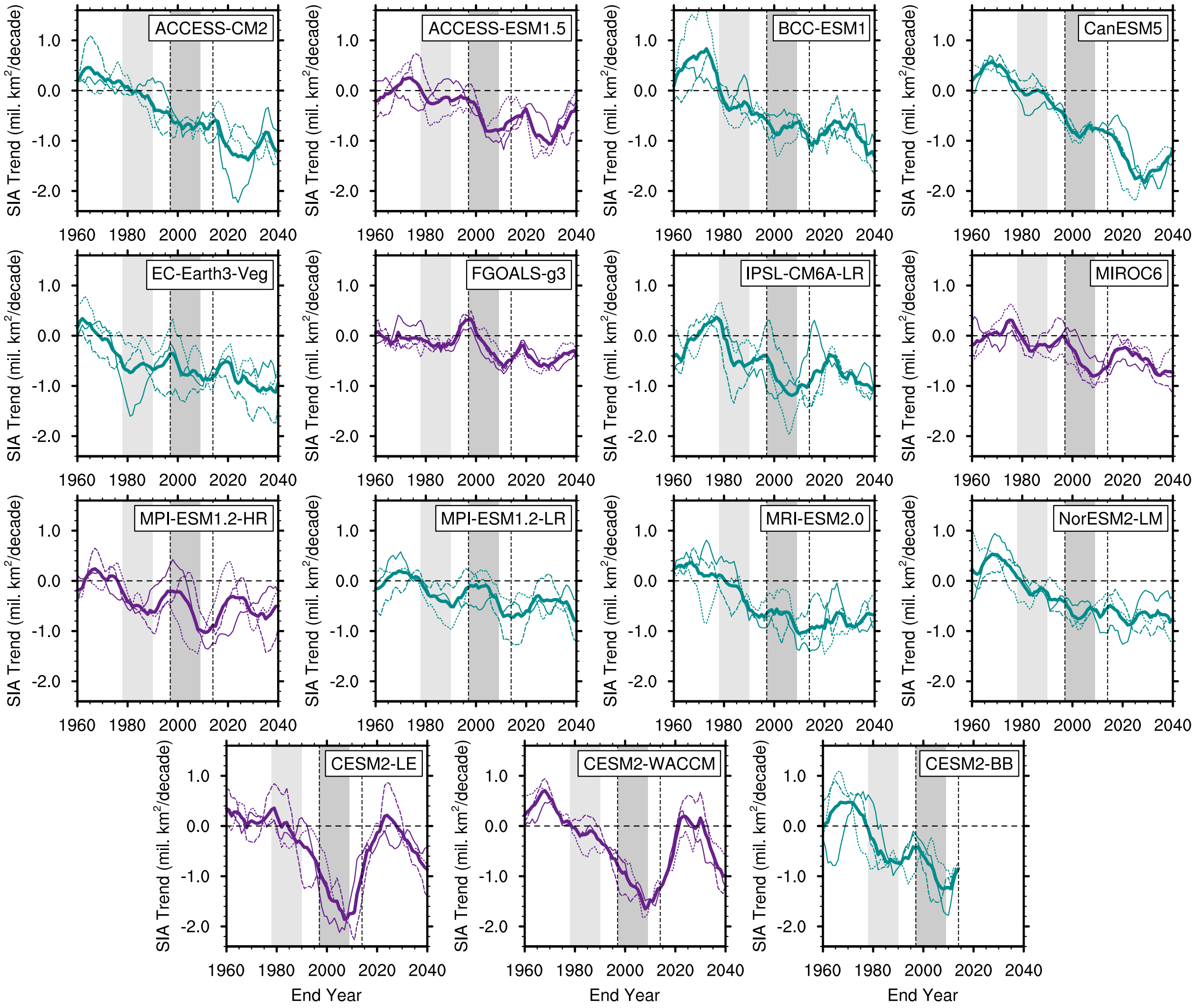

Fig. S7. September sea ice area trends in CMIP6 models. As in Fig. S6, but for 20-year linear trends in September sea ice area (SIA). The horizontal dashed line indicates no trend. Values on the x-axis indicate the end year of the 20-year period over which the linear trend is computed.
